# Supplementary material for: Modeling hepatocellular carcinoma and its microenvironment on a chip
Source: Cell Death Discov. 2025 Dec 29;12:55. doi: 10.1038/s41420-025-02917-8 (PMC12847976; doi:10.1038/s41420-025-02917-8)
Supplement: Supplementary file 1 — Supplemmental Information [file 41420_2025_2917_MOESM1_ESM.docx]

**Supplemmental Information**

**Modeling Hepatocellular Carcinoma and its microenvironment on a chip**

**Hepatocellular carcinoma PDChip**

Orsola Mocellin^1^, Stéphane Treillard^1^, Abbie Robinson^1^, Aleksandra Olczyk^1^, Thomas Olivier^1^, Chee P. Ng^1^, Arthur Stok^1^, Gilles van Tienderen^2^, Monique M.A. Verstegen^2^, Jeroen Heijmans^1^, Dorota Kurek^1^, Sebastian J. Trietsch^1^, Henriëtte L. Lanz^1^, Paul Vulto^1^, Jos Joore^1^ and Karla Queiroz^1,*^

^1^MIMETAS BV, De Limes 7, NL-2342DH Oegstgeest, The Netherlands

^2^Department of Surgery, Erasmus MC Transplant Institute, Erasmus MC-University Medical Center Rotterdam, NL-3015GD Rotterdam, The Netherlands

**Correspondence*: k.queiroz@mimetas.com

**Inventory of Supplemental Information**

**Supplementary Figure 1, is related to Figure 1**

**Supplementary Figure 2, is related to Figure 3**

**
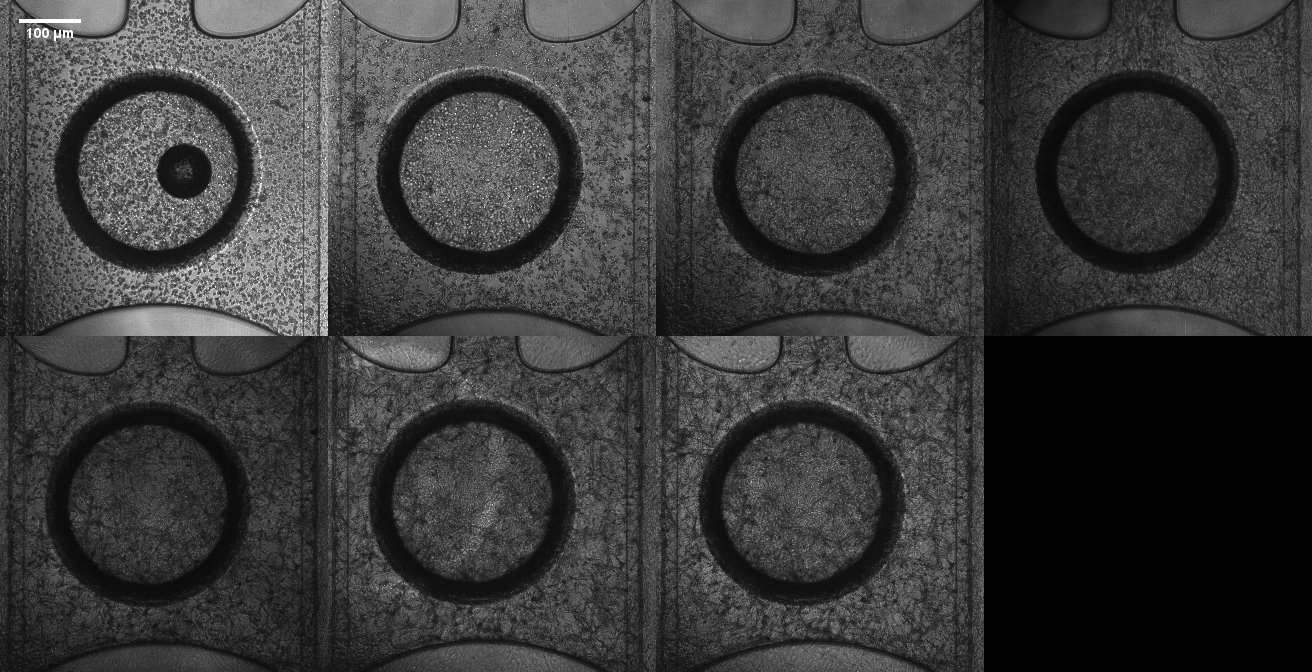
**

**D3**

**D1**

**D0**

**D6**

**D7**

**D9**

**D8**

**Supplementary Figure 1. Phase contrast images of HCC PDChips on day 6 and day 9**. HCC PDChips control condition show an organized vasculature at the start of the drug exposure experiment (Day 6) that remain stable throughout 72 hours (Day 9).

**HCC1**


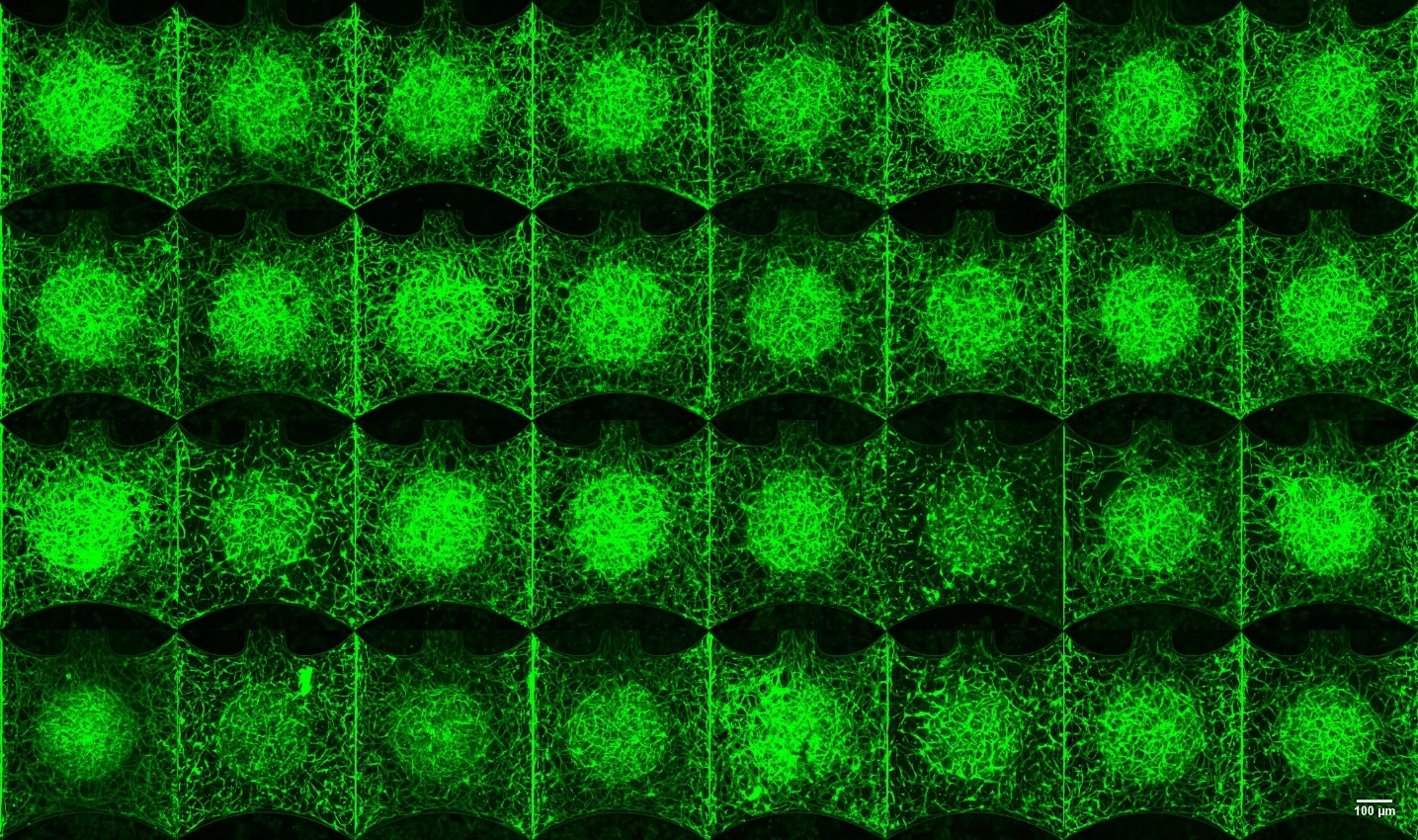

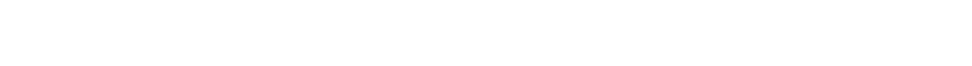

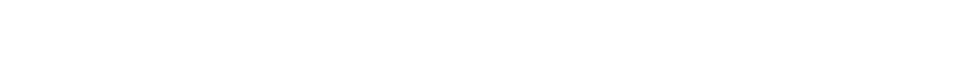

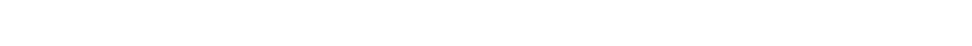

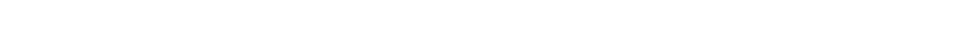


**HCC2**


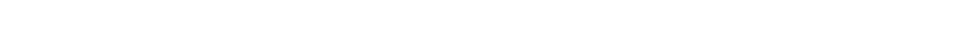

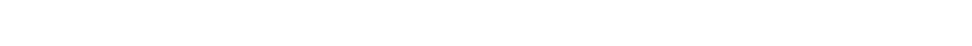

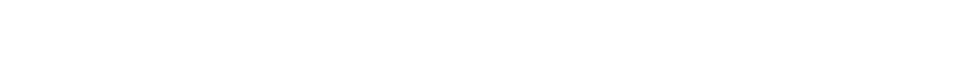

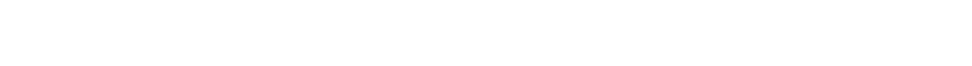


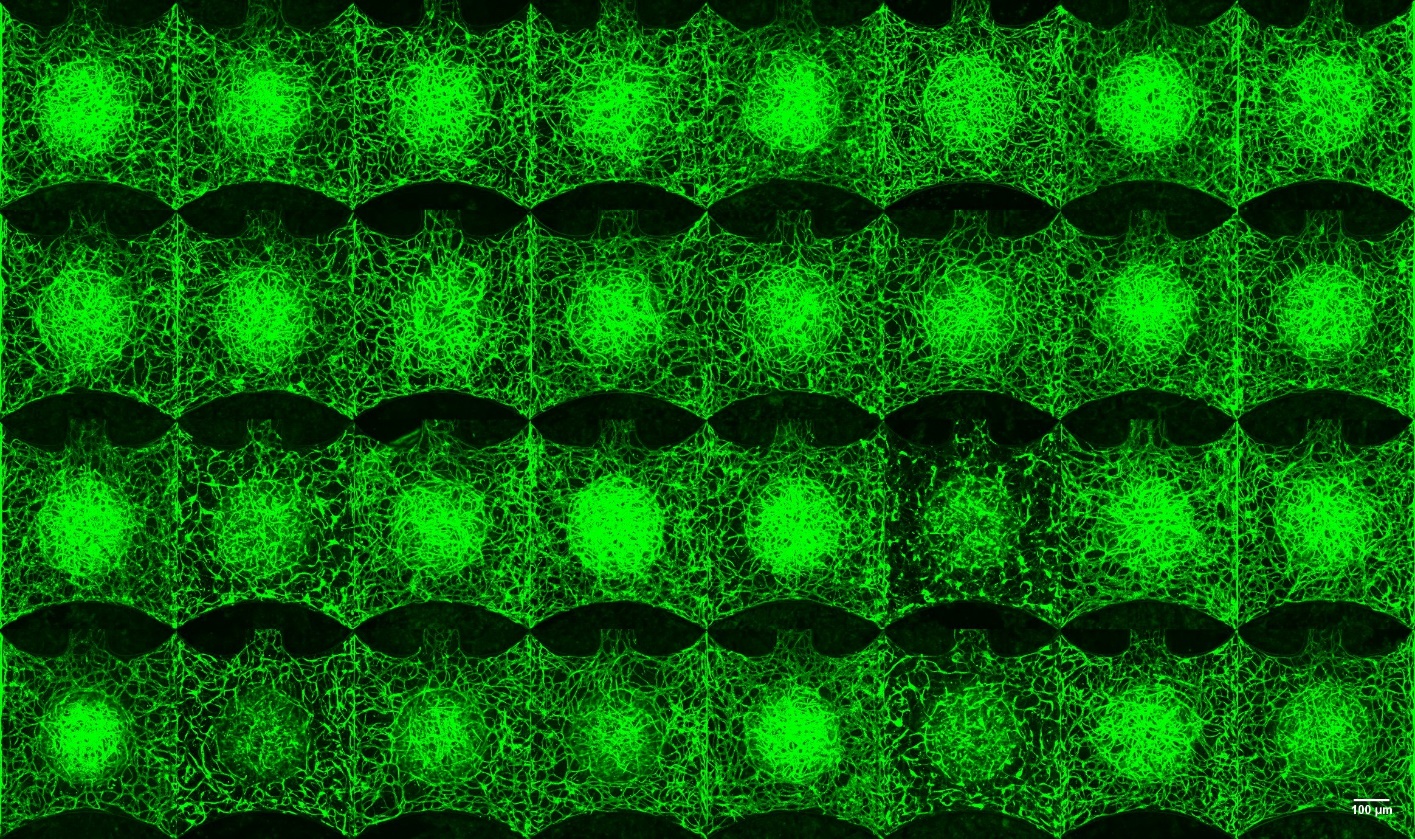


**HCC3**


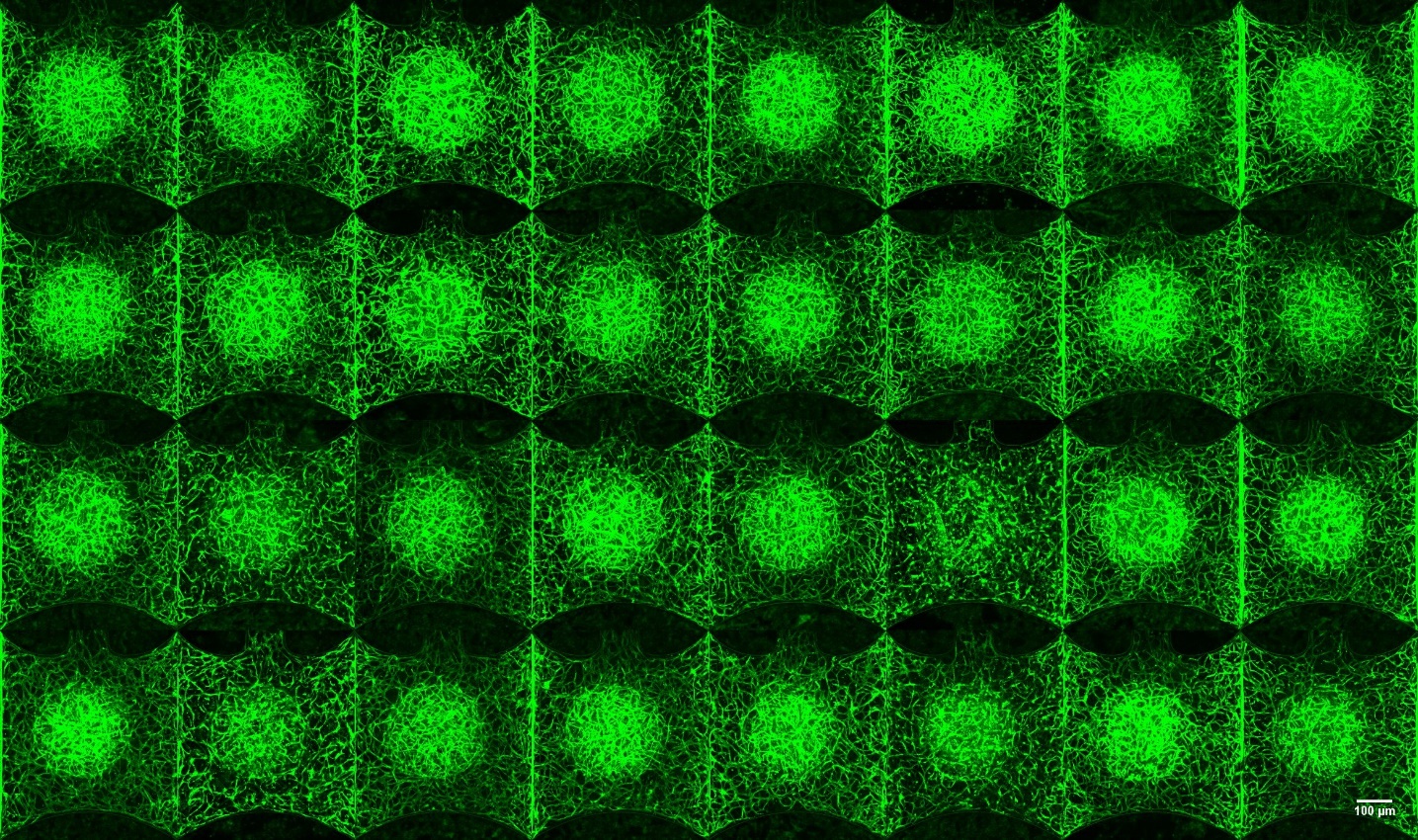

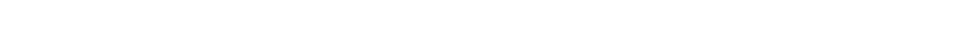

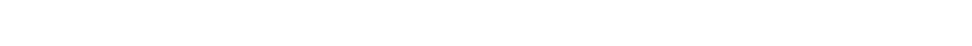

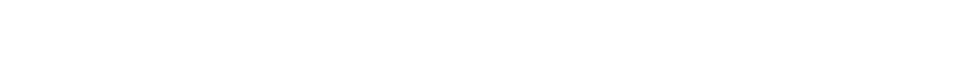

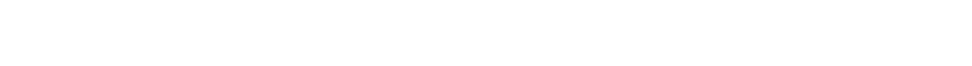


**HCC4**


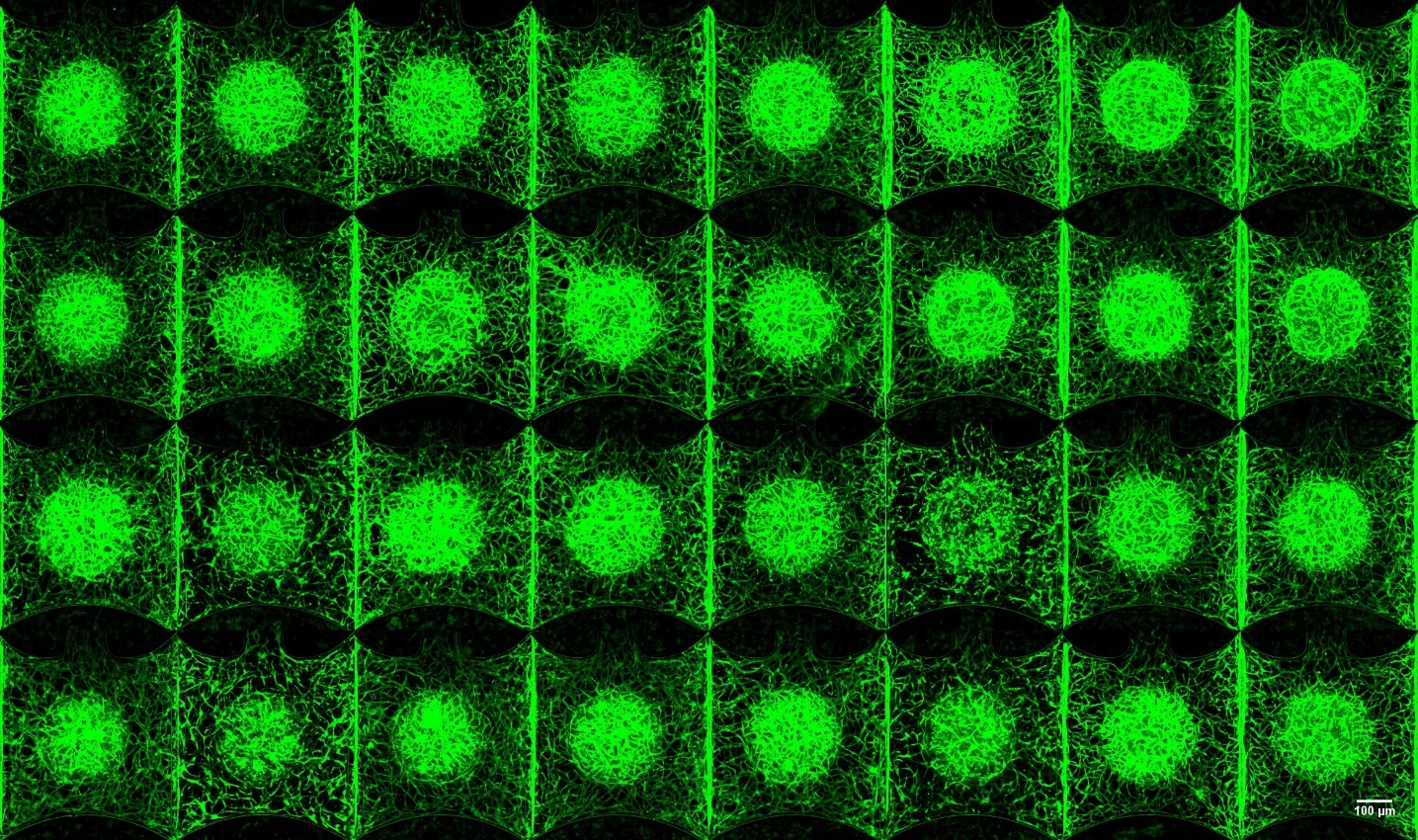

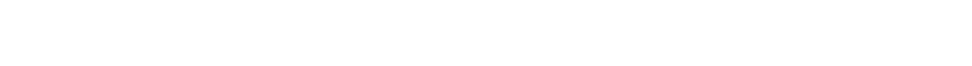

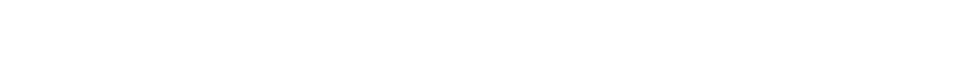

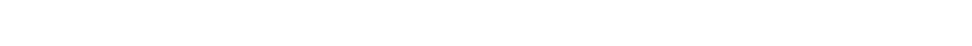

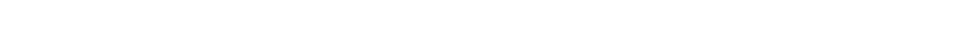


**HCC5**


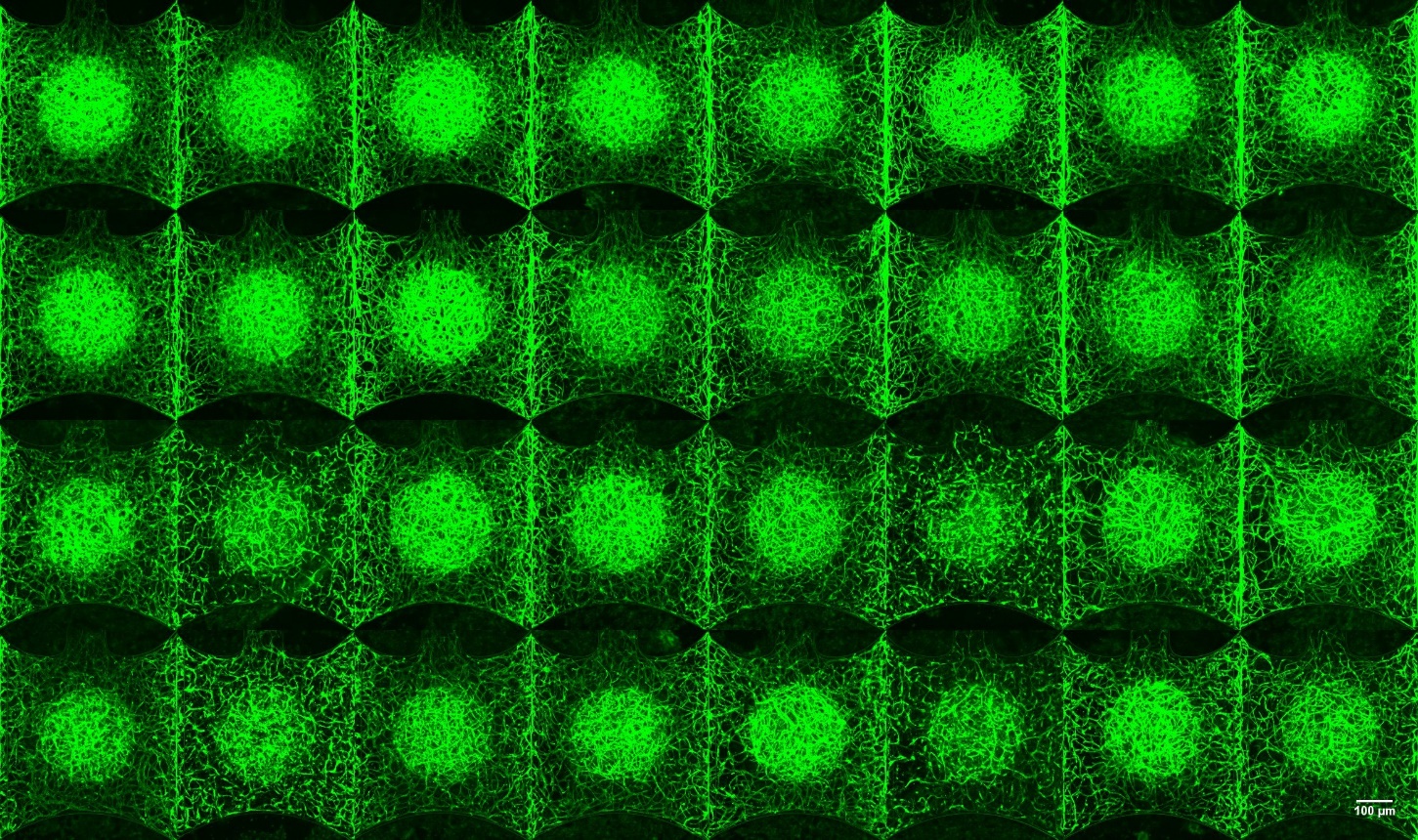

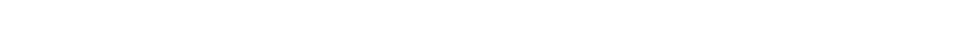

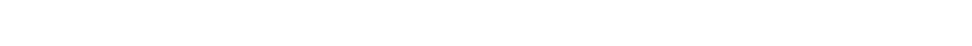

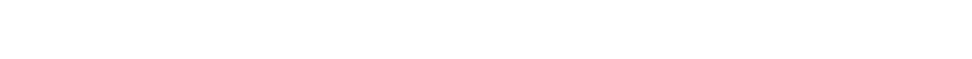

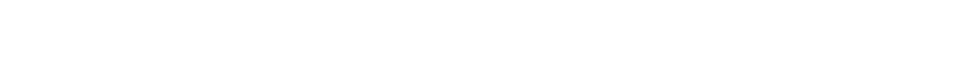


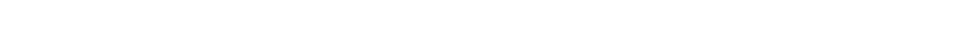

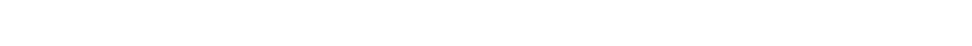

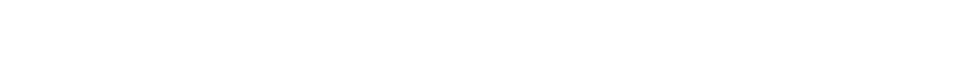

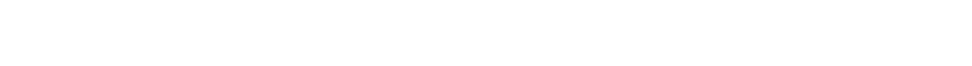


**HCC6**


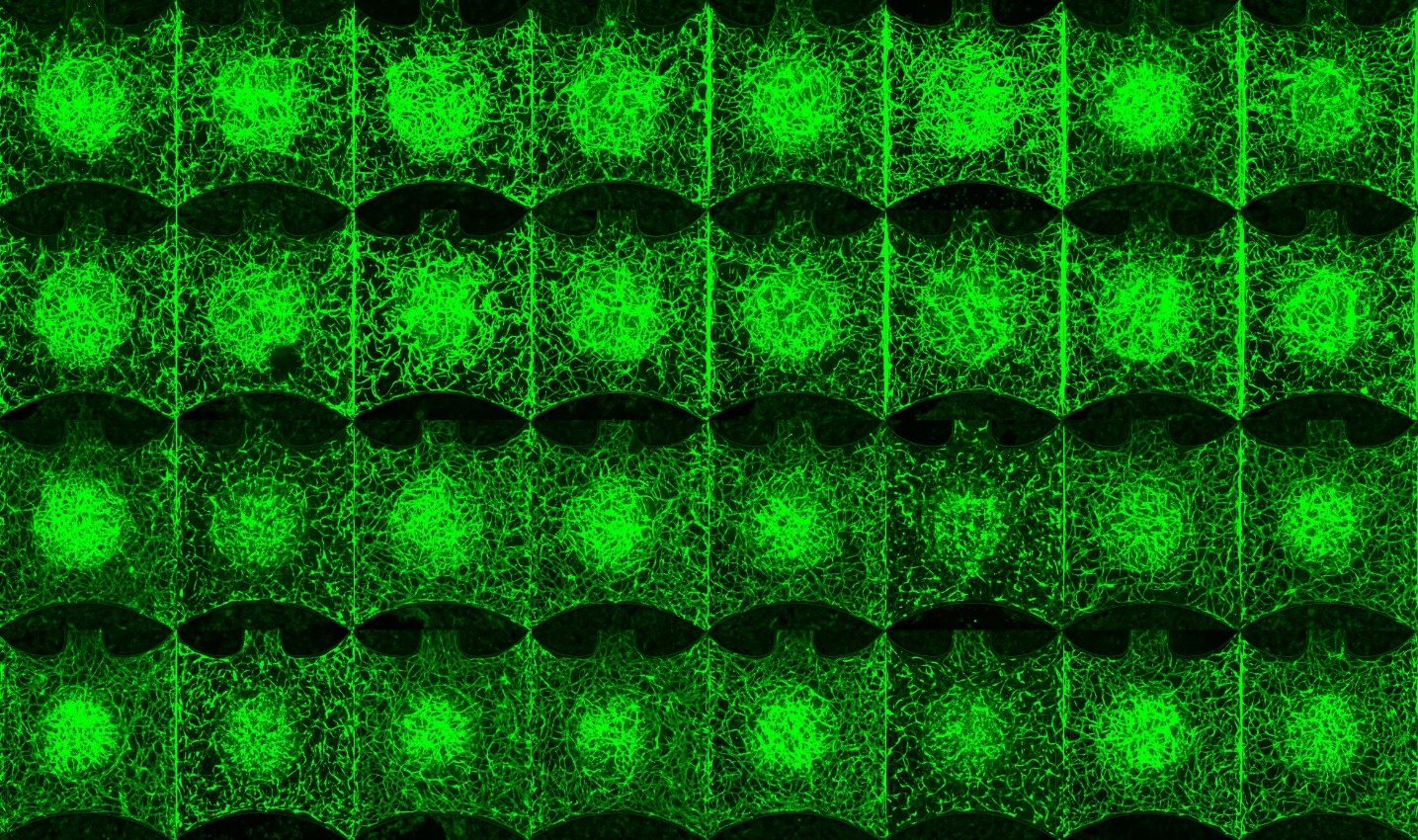

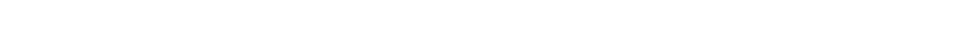

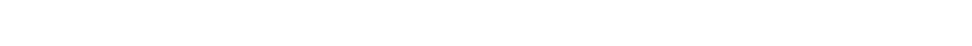

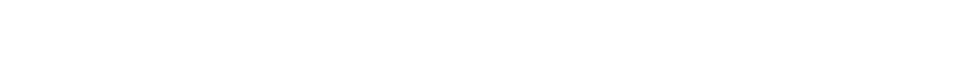

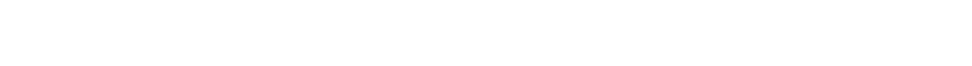


**HCC7**


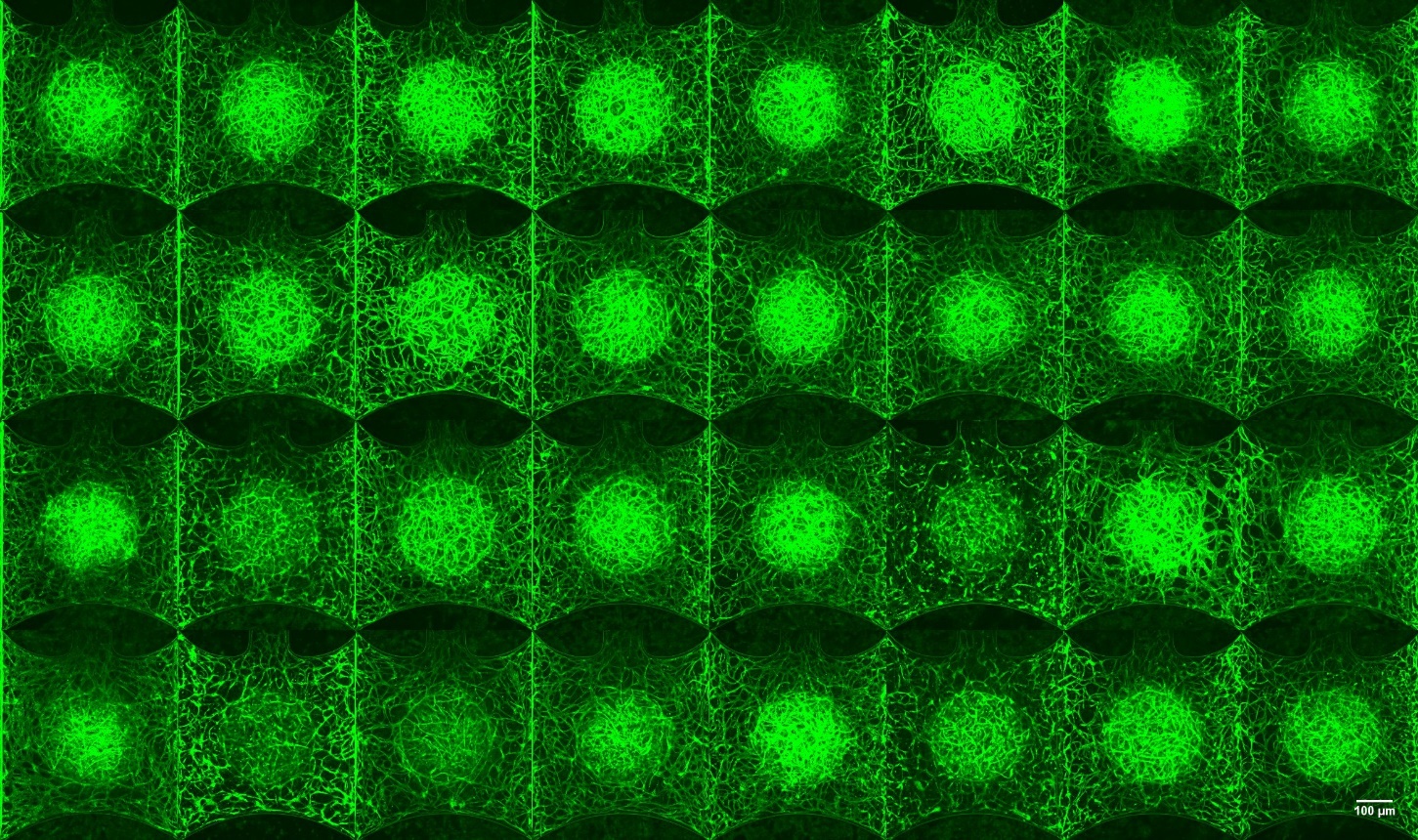

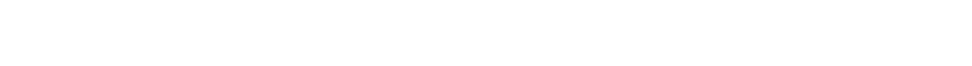

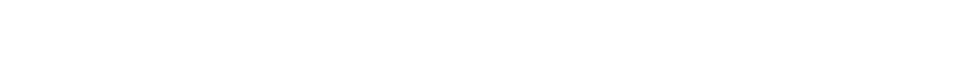

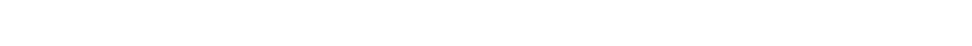

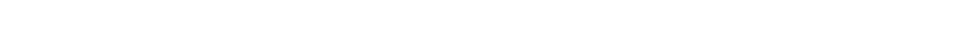


**HCC8**


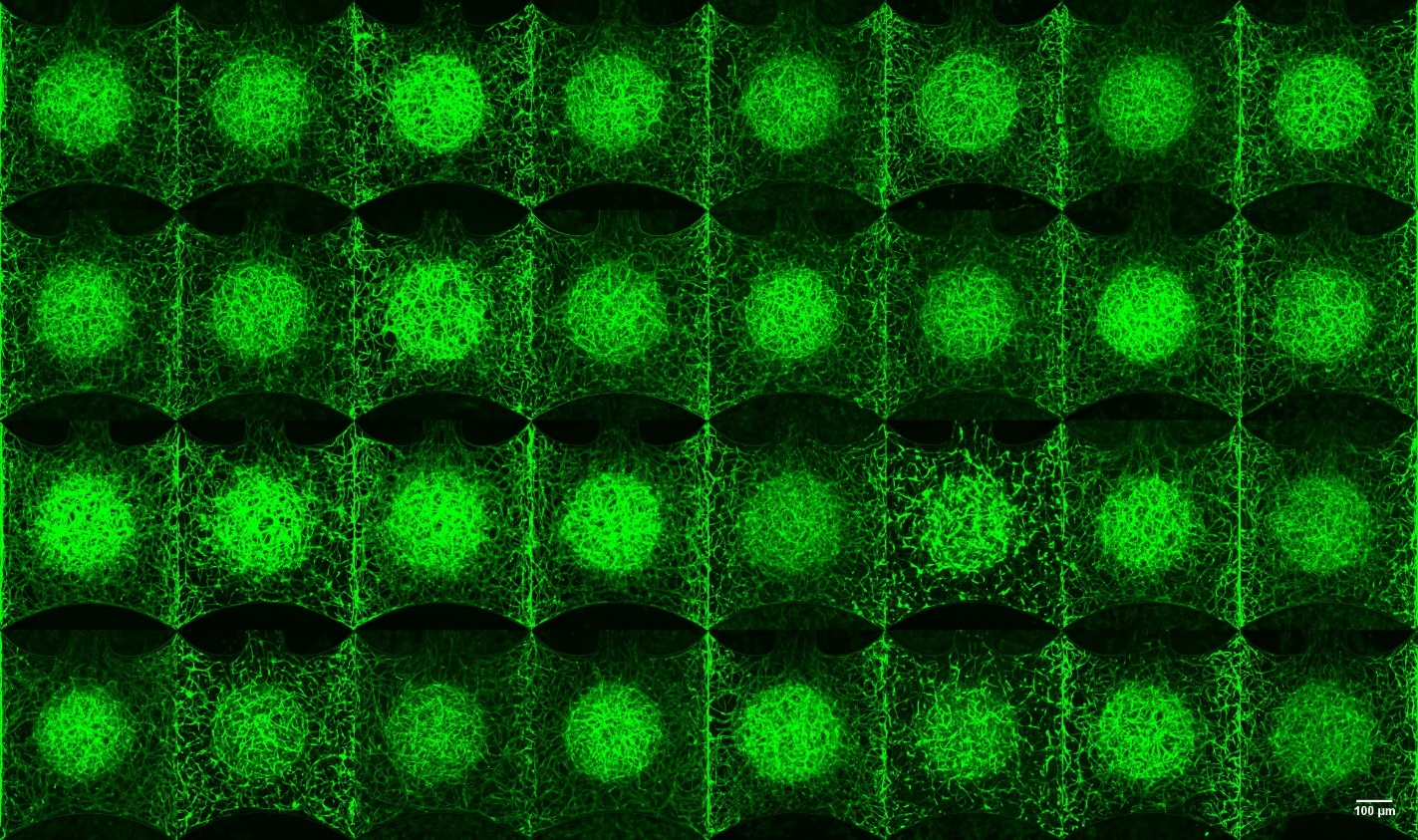

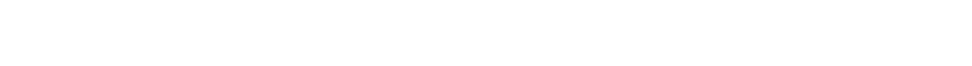

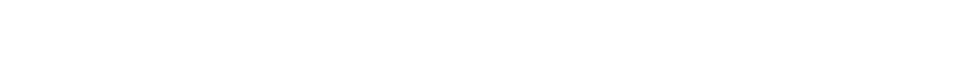

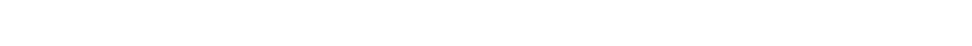

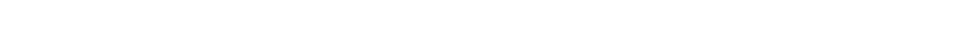


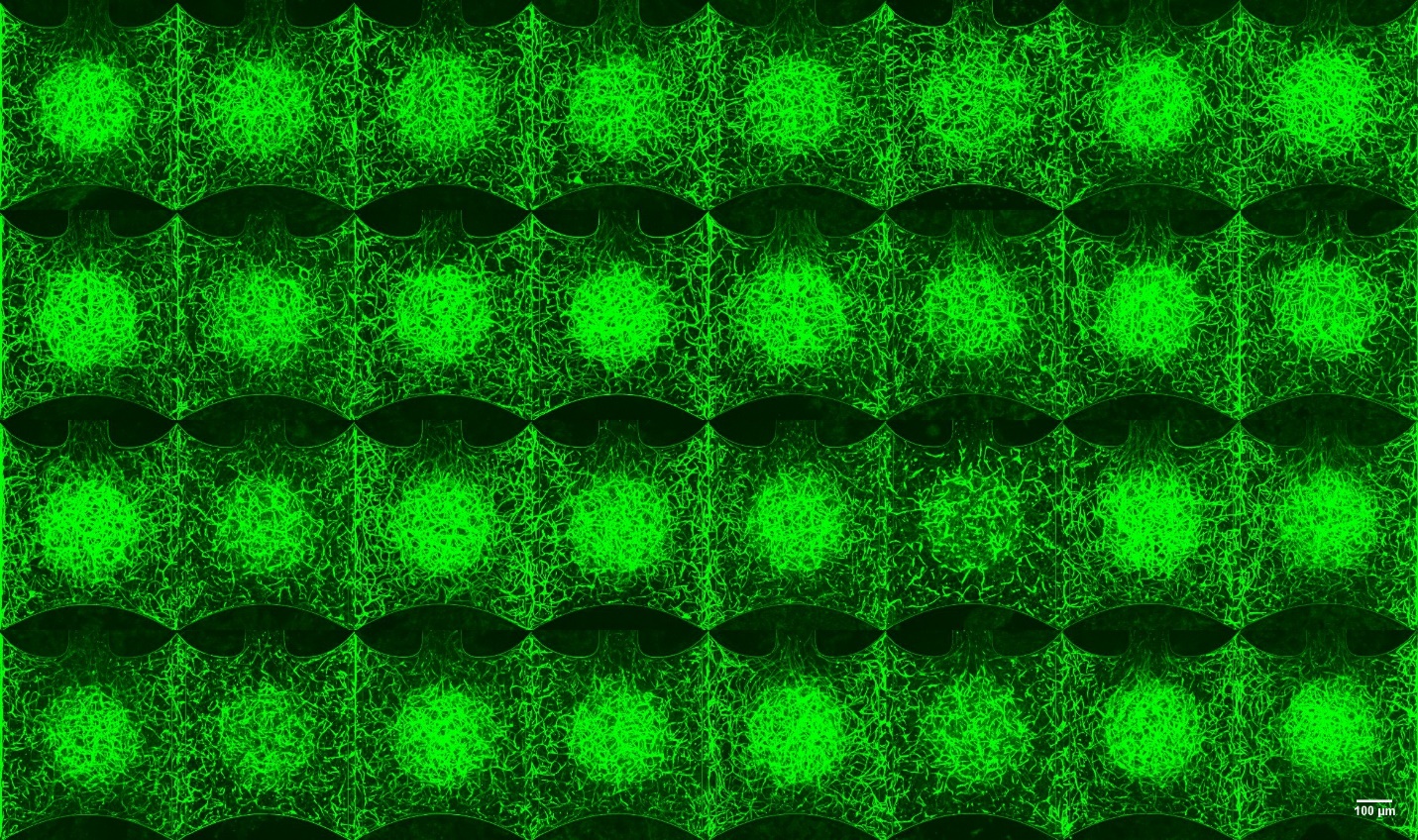

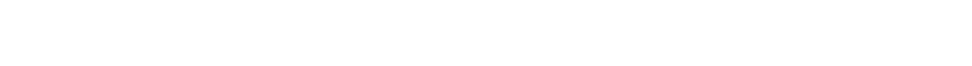

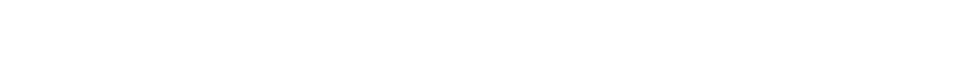

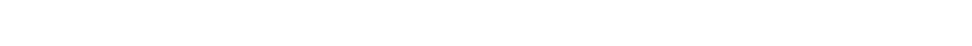

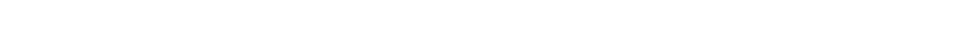

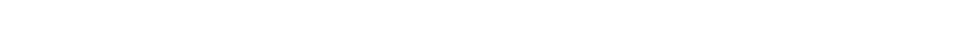

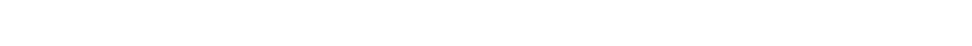


**Huh7**

**
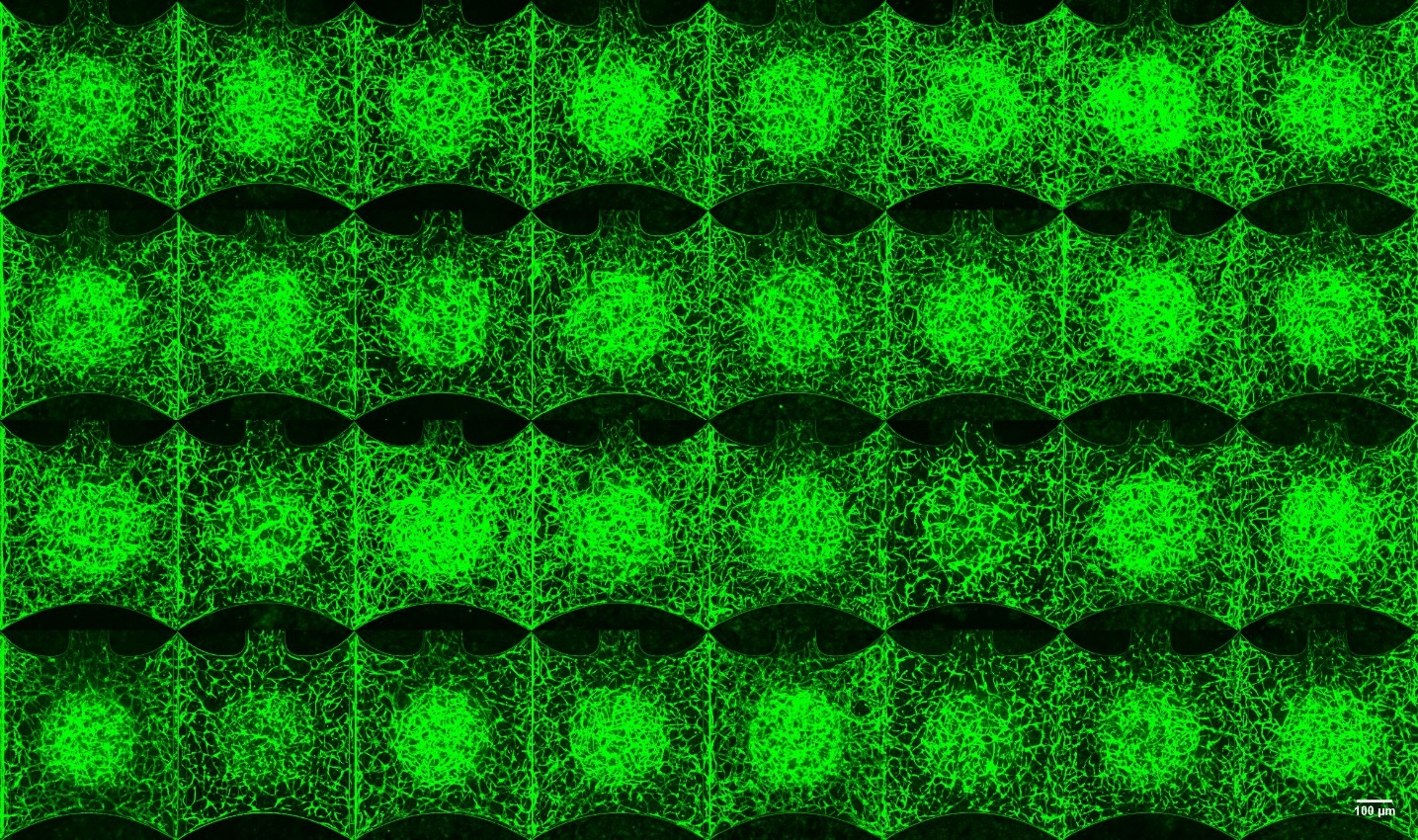
**

**HLE**


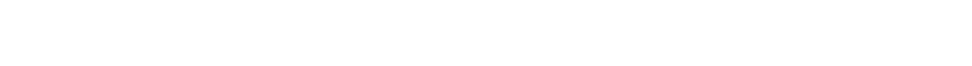

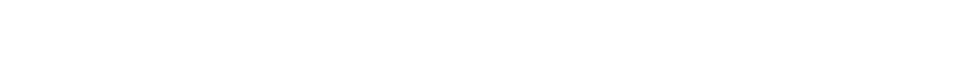

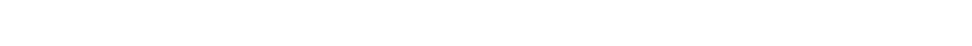

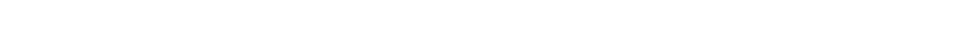


**Supplementary Figure 2. HCC PDChip-associated vasculature.** HCC vascularized constructs (HCC 1-8, Huh7 and HLE) were immunostained for CD31 (green) after drug exposure (72h). Immunostained HCC PDChips were confocal imaged and used for the phenotypical assessment of the vasculature. Abbreviation of drug names: Gal 0.1µM and 1µM (Galunisertib), Vac 0.1µM and 1µM (Vactosertib), LY 0.1µM and 1µM (LY2090314), WZ 1µM and 10µM (WZ811), Ator 5µM (Atorvastatin), SH 0.1µM and 1µM (SH-4-54), Sora 0.1µM and 1µM (Sorafenib), Atez 5µg/mL (Atezolizumab), Beva 5µg/mL (Bevacizumab), Halo 0.1µM (Halofuginone), UNB 10µM (UNBS5162), Lenv 0.1µM and 1µM (Lenvatinib), Toci 5µg/mL (Tocilizumab), Cri 0.1µM and 1µM (Crizotinib), Atez-Beva (Atezolizumab 5µg/mL-Bevacizumab 5µg/mL), Vac-SH (Vactosertib 0.1µM -SH-4-54 0.1µM), UNB-SH (UNBS5162 10µM -SH-4-54 0.1µM), WZ-SH (WZ811 1µM - SH-4-54 0.1µM), UNB-Halo (UNBS5162 10µM -Halofuginone 0.1µM), WZ-LY (WZ811 1µM - LY2090314 0.1µM). DMSO (Dimethyl sulfoxide 0.1%) , and IgG1 (Immunoglobulin G1 10µg/mL) were used as vehicle controls. Atezolizumab 5µg/mL, Bevacizumab 5µg/mL and Tocilizumab 5µg/mL, also contains IgG1 5µg/mL.
